# Human gut microbiota in health and disease: Unveiling the relationship

**DOI:** 10.3389/fmicb.2022.999001

**Published:** 2022-09-26

**Authors:** Muhammad Afzaal, Farhan Saeed, Yasir Abbas Shah, Muzzamal Hussain, Roshina Rabail, Claudia Terezia Socol, Abdo Hassoun, Mirian Pateiro, José M. Lorenzo, Alexandru Vasile Rusu, Rana Muhammad Aadil

**Affiliations:** ^1^Department of Food Science, Government College University Faisalabad, Faisalabad, Pakistan; ^2^National Institute of Food Science and Technology, University of Agriculture, Faisalabad, Pakistan; ^3^Department of Genetics, University of Oradea, Oradea, Romania; ^4^Sustainable AgriFoodtech Innovation & Research (SAFIR), Arras, France; ^5^Syrian Academic Expertise (SAE), Gaziantep, Turkey; ^6^Centro Tecnológico de la Carne de Galicia, Ourense, Spain; ^7^Área de Tecnoloxía dos Alimentos, Faculdade de Ciências de Ourense, Universidade de Vigo, Ourense, Spain; ^8^Life Science Institute, University of Agricultural Sciences and Veterinary Medicine Cluj-Napoca, Cluj-Napoca, Romania; ^9^Faculty of Animal Science and Biotechnology, University of Agricultural Sciences and Veterinary Medicine Cluj-Napoca, Cluj-Napoca, Romania

**Keywords:** human gut microbiota, health, disease, eubiosis, dysbiosis, pathogenic

## Abstract

The human gut possesses millions of microbes that define a complex microbial community. The gut microbiota has been characterized as a vital organ forming its multidirectional connecting axis with other organs. This gut microbiota axis is responsible for host-microbe interactions and works by communicating with the neural, endocrinal, humoral, immunological, and metabolic pathways. The human gut microorganisms (mostly non-pathogenic) have symbiotic host relationships and are usually associated with the host’s immunity to defend against pathogenic invasion. The dysbiosis of the gut microbiota is therefore linked to various human diseases, such as anxiety, depression, hypertension, cardiovascular diseases, obesity, diabetes, inflammatory bowel disease, and cancer. The mechanism leading to the disease development has a crucial correlation with gut microbiota, metabolic products, and host immune response in humans. The understanding of mechanisms over gut microbiota exerts its positive or harmful impacts remains largely undefined. However, many recent clinical studies conducted worldwide are demonstrating the relation of specific microbial species and eubiosis in health and disease. A comprehensive understanding of gut microbiota interactions, its role in health and disease, and recent updates on the subject are the striking topics of the current review. We have also addressed the daunting challenges that must be brought under control to maintain health and treat diseases.

## Introduction

The association of human health with the intestine has been long acknowledged as Hippocrates said, “Death sits in the bowls” in 400 B.C. Many studies worldwide have focused on the significant impact of intestinal microbiota on human health and disease (AboNahas et al., 2022). The human body is colonized by a diversity of bacteria, viruses, archaea, and unicellular eukaryotes. Microbes inhabit all human body surfaces, but a significant number of microbes live in the gastrointestinal tract/gut. The human gut possesses approximately more than one thousand microbial species that form a complex ecological community called gut microbiota (Lagier et al., 2016). The human gut microbiota is carrying about 150 times more genes compared to the entire human genome. It is widely accepted that approximately a hundred trillion microbes live on and inside the human body having a key role in various biological processes including health and disease (Wang et al., 2017). They are the primary mediators of body homeostasis, impacting various physiological activities, such as metabolism, barrier homeostasis, inflammation, and hematopoiesis through both intestinal and extra-intestinal actions. The gut microbiota has recently been classified as a “vital organ” because of its multidirectional and communicational connection or axis with other organs through neural, endocrine, humoral, immunological, and metabolic pathways. Any change in the microbial community not only causes gut-related issues but also influences other organs related diseases, though the actual interaction mechanism between the gut and the organs has yet to be fully understood (Ahlawat and Sharma, 2021).

The interaction between host and microbes plays a pivotal role in both health and disease. Gut microbiota diversity is greatly dependent on various host factors including diet, human lifestyle, age, and environmental factors. However, diet is currently considered one of the major factors (modifiers) in modulating the gut microbiota (Simões et al., 2022). Human microbiota has promising potential in altering appetite, increasing nutrient harvest, and exerting energy from various food components. Microbes have also a fundamental role in xenobiotic metabolism. In xenobiotic metabolism, various gut microbes alter the chemical structures of various diet components, drugs, pollutants, and many pesticides (Nakov and Velikova, 2020).

**FIGURE 1:**
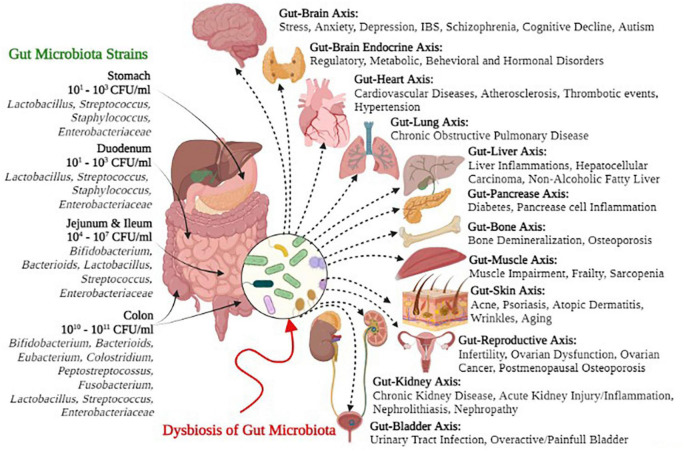
Gut microbial strains and negative health outcomes of gut microbial dysbiosis.

Many research studies have supported the concept that gut microbiota plays a key role in modulating immunity, weight gain or loss, energy homeostasis, and obesity-related disorders (Piccioni et al., 2022). Likewise, gut microbiota and their metabolites are associated with various non-alcoholic fatty liver diseases (NAFLDs), inflammatory bowels diseases (IBDs), hepatocellular carcinoma, cardiovascular diseases (CVDs), alcoholic liver disease (ALD), chronic kidney diseases (CKDs), and cirrhosis (Hsu et al., 2020; Jansen et al., 2021; Ryma et al., 2021; Wang et al., 2021; Zhou et al., 2021; Philips et al., 2022). Figure 1 depicts several symbiotic gut microbial strains and the possible negative health consequences of dysbiosis on the gut-organ axis. Hence, the comprehensive understanding of recent gut microbiota interactions, their eubiotic role in health and disease, and other recent updates on this subject are compiled in this review, with a major focus on controlling the challenges to maintain health and treat various diseases.

## Significance of human gut microbiota eubiosis

Comprehensive clinical studies are available on microbiota and involvement in their balance, i.e., eubiosis and related pathophysiological aspects. The compositional difference in gut microbiota has been observed in health and disease conditions. Eubiosis conditions are effective in controlling various diseases caused by microbes. Proper intake of a healthy diet and the development of eubiosis acts in favor of human health. The high intake of antibiotics causes an imbalance in the gut microbiota and favors systemic diseases (Santacroce et al., 2021).

Several population-based studies have revealed the highly beneficial role of human gut microbiota in healthy people, as well as the importance of well-understanding its structure and the factors that influence its composition, such as food, age, geography, systemic disorders, and drugs (Wang et al., 2017; Rowland et al., 2018). Phyla Firmicutes, Bacteroides, Actinobacteria, Proteobacteria, and Verrucomicrobia contribute to the significant resident bacterial populations in the gut microbiome (Fava et al., 2019). The first step in identifying the symbiotic interactions between intestinal microbes and their hosts is to describe the balanced composition of gut microbiota and disease-related variations. The microbes reside in a mutual association with the host in a healthy state, affecting the host’s health by controlling nutrient metabolism, defending against pathogens, and delivering signals to immune cells to promote host physiology and immunity (Ribaldone et al., 2022). An initial underestimation of the total number of microbial species in the intestine has been described through several *vivo* and *ex vivo* studies due to complications in culturing certain microorganisms (Lagier et al., 2015).

**FIGURE 2:**
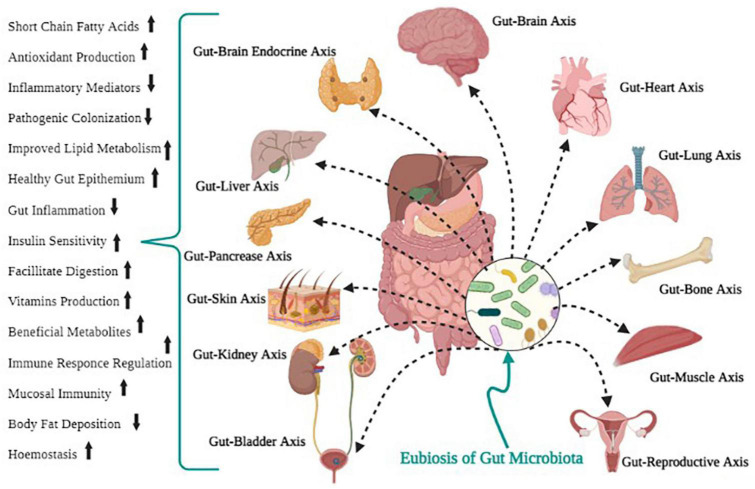
Positive health outcomes of gut microbial eubiosis.

Bacteria and proteobacteria contribute to carbohydrate digestion, gut microbiota, regulation of the immune system, and defense against pathogen colonization (Rosser and Mauri, 2016; Fan and Pedersen, 2021). For survival, microbes in the intestine tract mainly depend on dietary substrates undigested in the upper digestive tract. Saccharolytic bacterial fermentation typically creates advantageous metabolites, while bacteria switch to an alternative energy source if there are insufficient carbohydrates, leading to the development of other metabolites that could be more disadvantageous to human health (Rowland et al., 2018). *Methanobrevibacter smithii* is the human-associated Archaea that plays a vital function in the synthesis of methane from H2 processed by bacterial metabolism. It is a prominent and essential Archean in the gut microbiota (Hoffmann et al., 2013; Berry, 2016). Some of the beneficial functions of gut microbiota for human health are shown in Figure 2.

It is considered that diet is a significant factor associated with health and disease control, but some recent studies concluded that diet is pivotal for shaping the gut microbial structure and influencing the metabolism of the host. The gut environment, sequentially, can help reproduce, grow, and survive the microbial community (Browne et al., 2016). Carbohydrates are an essential and significant energy source; also, intestinal microbiota has provided a fermentation stage to deliver vital biomolecules to the host (Conlon and Bird, 2015).

A normal balance between the host and gut flora is essential for human health, while disruption is linked with various human diseases, like hypertension, obesity, cardiovascular disorders, diabetes, and IBD (Von Martels et al., 2017; Kho and Lal, 2018; Szablewski, 2018). However, the human microbiome analysis is still at its initial phase in filling the knowledge gap in the microbiome-host relationship and its role in disease pathogenesis and therapeutical importance. Therefore, further in-depth research is needed to unravel this fascinating yet enigmatic area of study.

### Gut microbiota and human metabolism

The diverse human microbiome has substantial metabolic activities essential for the functioning of mammalian enzymes in the gut mucosa and liver and the host metabolism. Gut microbiota influence host health by shaping the biochemical profile of the diet. The significant role of gut microbiota in human immunity has promoted research to investigate the contributions of particular microbes in metabolic pathways, especially in dietary components’ metabolism (Cardona and Roman, 2022). Recent studies have found that gut microbiota can metabolize phytochemicals, especially polyphenols, by well-defined paths (Rowland et al., 2018). The human gut microbiota reacts efficiently to major dietary changes. The presence of these fast, diet-induced patterns is confirmed by evidence from individuals switching between plant and meat-based diets, adding to their diet more than 30 g of specific dietary fibers a day or adapting either a high-fiber-low fat diet or a low-fiber-high-fat diet for ten days; in all cases, the structure and composition of microbiome changed over 1–2 days (Wu et al., 2011; David et al., 2014). This flexibility may be an advantageous feature of enlisting microbes as part of the digestive structure, particularly when considering the potential day-to-day variability in food available to foragers. It may also be an inescapable consequence of dealing with such a microbial community that is diverse and competitive and undergoes rapid turnover. Human gut microbiota is associated with the degradation of dietary fibers, proteins, and peptides by fermentation and anaerobic degradation (Yadav et al., 2018).

Carbohydrates and simple sugars are the main components of food metabolized by gut microbiota. Bacterial species, especially the phyla Bacteroidetes and Firmicutes, can ferment fibers (the indigestible carbohydrates) to produce branched-chain and short-chain fatty acids (SCFAs), lactate, ethanol, hydrogen, and carbon dioxide; these products are further used by the host or excreted (Patrascu et al., 2017). Acetate, propionate, and butyrate are the main short-chain fatty acids (SCFAs) distinguished in human feces, usually found in 3:1:1 to 10:2:1 molar ratio; this ratio is consistent with the values reported in the intestine in early sudden deaths (Rowland et al., 2018). These are the main SCFAs that perform several essential functions in the human body (Rauf et al., 2022). Butyrate is perhaps the essential SCFA for human health, as it is the primary source of energy for human colonocytes (Wang et al., 2019). Butyrate has the potential to act as an anti-carcinogen as it persuades apoptosis of colon cancer cells and regulates gene expression by inhibiting histone deacetylase (Havenaar, 2011; Steliou et al., 2012). Propionate is also an essential energy source for the epithelial cells in the liver; it plays a vital role in gluconeogenesis (Cani, 2018). Acetate helps in the growth of other bacteria as an essential co-factor; for example, *Faecalibacterium prausnitzii* will not grow in pure culture in the absence of acetate (Rowland et al., 2018).

Human gut microbiota can also synthesize essential vitamins, including biotin, folate, and vitamin K, and neutralize carcinogenic compounds, such as pyro lysates (Selber-Hnatiw et al., 2017). Various indications specify that the host metabolism is mainly affected by multiple microbial metabolites that bind to specific host membranes or nuclear receptors (Bhutia et al., 2017). Some of the most important metabolites produced by gut microbiota are described in Table 1. The majority of essential functions for host physiology and maintenance are associated with gut microbiota, e.g., the nervous system’s development, intestinal development, appetite regulation, etc.

**TABLE 1:** Metabolites produced by gut microbiota and their functions.

Metabolites	Functions	References
Bile acid metabolites; including deoxycholic acid (DCA) and lithocholic acid (LCA)	Regulate bile acid, cholesterol, lipid, glucose, and energy metabolism, show antimicrobial effects, and activate host nuclear receptors and cell signaling pathways.	Ramírez-Macías et al., 2022
Short-chain fatty acids (SCFAs) metabolites such as propionate and butyrate	Regulate food intake and insulin secretion, also aid in maintaining body weight.	Psichas et al., 2015; Larraufie et al., 2018
Branched-chain fatty acids (BCFA) including isobutyrate, isovalerate	Histone deacetylase (HDAC) inhibition, increased histone acetylation.	Mischke and Plösch, 2016
Indole derivatives including indoxyl sulfate and indole-3-propionic acid (IPA)	IPA exhibits neuroprotective effects, acts as a powerful antioxidant, and regulates intestinal barrier function. Indoxyl sulfate is a uremic toxin that accumulates in the blood of individuals with impaired excretion systems.	Hendrikx and Schnabl, 2019
Lipopolysaccharide (LPS), peptidoglycan (PGN), lipoteichoic acid (LTA)	Epigenetic regulation of genes in colorectal cancer, modulation of chromatin structure and transcriptional activity.	Lightfoot et al., 2013; Mischke and Plösch, 2016
Phenolic derivatives include 4-OH phenylacetic acid, urolithins, enterodiol, and 9-prenylnaringenin	Exhibit antimicrobial effects, maintain intestinal health, and protect against oxidative stress.	Larrosa et al., 2010
Choline metabolites include choline, trimethylamine N-oxide (TMAO), and betaine	Regulating lipid metabolism, and glucose synthesis contribute to the development of cardiovascular disease.	Smallwood et al., 2016
Polyamines include putrescine, spermidine, and spermine	Sustaining the high proliferation rate of intestinal epithelial cells enhances intestinal barrier integrity and enhances the systematic adaptive immune system.	Rooks and Garrett, 2016; Tofalo et al., 2019
Vitamins including thiamine (B1), riboflavin (B2), niacin (B3), pyridoxine (B6), pantothenic acid (B5), biotin (B7), folate (B11-B9), cobalamin (B12), and menaquinone (K2)	Help in red blood cell formation, DNA replication, and repair, work as an enzymatic co-factor, and enhance immune functioning.	Nicholson et al., 2012; Forster et al., 2017
Ethanol	Protein fermentation metabolites may be involved in NAFLD progression.	Yao et al., 2016; Wu et al., 2021
Hydrogen sulfide (H2s)	Reduction/neutralization of reactive oxygen species.	Afanas’ev, 2014; Mischke and Plösch, 2016

## Gut microbiota in immune homeostasis

The contribution of the human gut microbiota to various aspects of human health, especially the immune system, is crucial for providing the host with several essential benefits. Recent studies have found that early development of the gut microbiota is crucial in preventing autoimmune disorders and proper immune functioning (Lazar et al., 2018; Spencer et al., 2019; Elmassry et al., 2020; Schluter et al., 2020). The intestinal microbiome is essential for the maturation of the immune system, which includes adaptive and innate immune responses. Innate immunity deals with the physical barrier of the epithelia, specialized cells, and circulating chemicals to immediately identify a wide assortment of foreign antigens and eradicate them (Thaiss et al., 2016). The mucosal immune system, in particular, mechanisms are primarily independent of the systemic immune system, and after bacterial colonization of the intestinal tract, it undergoes significant changes. For the immune system’s growth and development, commensal microorganisms are necessary to distinguish between commensal and pathogenic bacteria. Recent studies have demonstrated that gastrointestinal tract microbiota modulates the movement and role of neutrophils and influences the division of populations of T cells into various forms of T helper cells (Th), respectively: Th1, Th2, and Th17 or into regulatory T cells (Francino, 2014; Owaga et al., 2015; Tomkovich and Jobin, 2016). Th17 cells are a subset of TCD4+ cells that secrete several cytokines, affecting immune homeostasis and inflammation (Rossi and Bot, 2013). Gut microbiota contributes to the stimulation and maturation of the immune system in response to pathogens, and it induces and sustains tolerance (Pickard et al., 2017).

Development of the immune system begins at birth, with the introduction of the microbiota, and can only become fully mature in the presence of commensal microflora. Proper immune system maturation is needed to prevent aberrant immune responses, which can cause chronic inflammation and illness (Tibbs et al., 2019). Various strategies, including the germ-free (GF) model, have been taken to demonstrate the importance of gut flora for forming both innate and adaptive immune systems (Uzbay, 2019). In comparison, gut microbiota modulation with antibiotic treatment also demonstrated its importance for immune homeostasis (Hill et al., 2010; Ubeda and Pamer, 2012). Antigen-presenting cells (APCs), having co-evolved with gut microbiota, a key advantage of intestinal APCs is their potential to defend the body from infection while retaining the immune tolerance to the normal gut microbiota (Wu and Wu, 2012). Gut microbiota plays a significant role in controlling the production of APCs. Gut microbiota is also involved in various intestinal and extraintestinal autoimmune diseases, as demonstrated by multiple studies (Andréasson et al., 2016; Rinninella et al., 2019).

## Gut microbiota in malnutrition and fasting

Diets and food supplements have a significant influence on the gut’s microbial composition and its variability over time. A high-fat diet is a risk factor for diseases like obesity, metabolic syndrome, and diabetes, all of which are linked to significant gut microbiota composition changes. Disruption of the circadian physiological rhythm increases the probability of intestinal dysbiosis, potentially leading to the pathogenesis of a variety of metabolic and inflammatory disorders, like diabetes, intestinal inflammatory diseases, and even cancer (Reynolds et al., 2017). Studies have also found that gut microbiota responds to malnutrition and fasting (Flint et al., 2015). The impacts of malnourishment on the gut microbiota were only studied under controlled conditions in lab animals due to ethical reasons. In a study, several weeks of nutrient deficiency showed increased microbiome diversity in fish, mice, and toads; geckos showed a decrease while no change was detected in quails (Kohl et al., 2014). Due to these variations, it is challenging to investigate the influence of human nutrient deficiency, which can only be experienced in particular undernourished people. One of the leading causes of child mortality is malnutrition; nutrient-rich therapeutic foods are used to treat severe malnutrition. Also, children cannot completely recover from body mass improvements, probably due to their immature microbiomes. In children, the early development of the intestinal microbiome is particularly significant because microbiome composition keeps changing as they grow and continue changing their diet (Derrien et al., 2019).

Weight loss is promoted by intermittent fasting (IF) regimens, which contribute to enhanced metabolic health. Through metabolic activities, IF participates in the modulation of the gut flora, allowing ongoing interaction with nutrients to be digested and shaping intestinal immune responses during the development of coronary heart disease, blood pressure, and diabetes mellitus (Matías-Pérez et al., 2022). Microbiota reshaping by antibiotic therapy has extended the survival of children with acute malnutrition; even so, severe malnutrition reappeared when the microbiome remained immature, implying that microbiota maturity would anticipate the long-term therapeutic efficacy of the food (Subramanian et al., 2014). Furthermore, a study found that gut microbiota contributes a beneficial impact to the start of severe malnutrition, which can be regenerated by microbiota transplantation into gnotobiotic mice (Smith et al., 2013). Dietary and lifestyle activity such as fasting, and time-restricted eating influences the makeup of the intestinal microbiota. Various microbial products such as SCFAs, trimethylamine N-oxide, tryptophan, and tyrosine derivatives can significantly change with significant microbiota composition changes. However, there are several promising observational studies on human malnutrition, holding out the hope that therapeutic renovation of the gut microbiota will support eradicating mortality linked to malnutrition.

## Gut microbiota in major human diseases

From the findings of recent epidemiological, physiological and omics-based studies, supported by cellular and animal experiments, it is demonstrated that intestinal microbiota plays a significant role in both health and disease (Ding et al., 2019). Although this research area is still at a very initial stage, with less understanding of the functional characteristics of the complex gut microbiota, some promising studies have been reported and indicated an enormous potential for revolutionizing the pathogenesis of diseases and therapeutic approaches (Ding et al., 2019; Yin et al., 2019; Rajoka et al., 2020; Bangar et al., 2022). Several major human diseases are associated with an altered gastrointestinal microbiota, for example, obesity, diabetes, cardiovascular disorders, cancer, hypertension, and IBDs (Ding et al., 2019; Nie et al., 2019; Xu et al., 2019) have been discussed individually later in this review. A state called “dysbiosis” is the variation in gut microbiota composition, which is described in many diseases, as shown in Table 2. It is a common problem in the current era because of bacterial infections, diet shifts, and antibiotics (Lindell et al., 2022). It has been challenging to define an appropriate healthy microbiome composition because of inter-individual variation (Lloyd-Price et al., 2016). A well-balanced gut microbial community is essential for the host and the microbiome to co-exist in a mutually beneficial relationship.

**TABLE 2:** Diseases associated with gut microbiota abnormalities.

Disease	Features	References
Irritable bowel syndrome	An abundance of Firmicutes and a decrease in Bacteroidetes.	Kennedy et al., 2014
Type 1 diabetes	In genetically predisposed individuals, autoimmune against pancreatic b-cells. Deficient development or alteration of the microbiota may contribute to dysfunctional immunity with the devastation of autoimmune b-cells and increased leakiness of the intestinal epithelial barrier. Variability of microbiomes reduced.	Dunne et al., 2014
Asthma	Outbreaks of *Chlamydophila pneumonia* during bronchitis and pneumonia development affect the airway microbiome. Gut microbiota is influenced by the introduction of microbiota to the environment, particularly in early life, which helps immune function growth and the development of defending against allergic sensitization.	Huang and Boushey, 2015
Food-borne pathogens and food poisoning	Opportunistic pathogens (*Campylobacter*, *Salmonella*, *Escherichia coli*, *Shigella*, etc.) disturb the microbiome’s balance leading to dysbiosis.	Josephs-Spaulding et al., 2016
Malnutrition	Decrease or missing species that either process food categories efficiently or produce vitamins may reduce the absorption of nutrients. An overabundance of *Enterobacteriaceae* can lead to epithelial damage, diarrhea, and limited absorption of nutrients.	Kane et al., 2015
Depression	In physiological systems, *Bifidobacterium infantis*, generally found in infants’ gastrointestinal tract and administered probiotic drugs, can have antidepressant effects.	Evrensel and Ceylan, 2015
Anxiety	Oral administration of *Campylobacter jejuni* subclinical doses in murine models induced anxiety-like behavior without stimulating immunity. In a marine model, the *Lactobacillus* and *Bifidobacterium* may act as an anxiolytic influencer.	Schnorr and Bachner, 2016

### Obesity

The global prevalence of obesity has exceeded nearly 650 million people in the last four decades, a total that is six times more than what was reported in the 1990s (Sørensen et al., 2022). That can only be justified by increasing caloric intake and decreasing physical activity (Pascale et al., 2019). Several other diseases, such as diabetes mellitus, coronary heart disease, and cancers, are linked to obesity (Amin et al., 2019; Sun et al., 2019). Thus, weight management and reduction have gained more interest and attention from researchers. The involvement of gut microbiota in obesity is becoming a broad research topic and potentially useful for obesity treatment. Remarkably, the effect of diet on intestinal microbiota composition has become a specific subject of research. In this regard, recent evidence from various studies of humans and mice has demonstrated that changes in gut microbiota composition may play a vital role in the development of obesity (Davis, 2016; Bouter et al., 2017; Stephens et al., 2018; Socol et al., 2022). Several gut microbiota species, called the obesogenic gut microbiota, can significantly contribute to obesity, such as *Firmicutes, Bacteroidetes*, *Rhizobium, Lactococcus*, and *Clostridium* (Cao et al., 2019). In particular, obesogenic gut microbiota could facilitate obesity by producing SCFAs such as butyrate, providing the host with extra energy, and inducing low-grade inflammation caused by intestinal microbiota metabolites (Cao et al., 2019). Genetic aspects and epigenetic variations also play a significant role in the correlation between the composition of the gut microbiota and its contribution to obesity and the production of metabolites.

Some mechanisms have been proposed to define the role of gut microbiota in the development of obesity. Gut microbiota can reduce fatty acid oxidation by suppressing adenosine monophosphate kinase (AMPk) (López, 2017). This enzyme is present in muscle fibers and the liver and serves as a cellular energy indicator. AMPk suppression leads to reduced oxidation of fatty acids and, as a result, increased fat accumulation. By inducing systematic inflammation, intestinal microbiota can also lead to metabolic disturbance observed in obesity (Pindjakova et al., 2017). Another proposed mechanism is the energy regulation and microbes’ potential to ferment dietary polysaccharides that are not digested by humans (Khan et al., 2016). The fermentation of dietary fiber produces SCFAs. SCFA can stimulate lipogenesis after being absorbed and boost triglyceride storage *via* molecular pathways. Also, SCFA has the potential to suppress the fasting-induced adipocyte factor (FIAF), which inhibits lipoprotein lipase (LPL), causing the accumulation of triglycerides in the host adipocytes (Khan et al., 2016). To acknowledge, how intestinal microbiota promotes the development of obesity, more prospective and interventional studies are needed.

### Hypertension

Hypertension is becoming a significant threat to public health and an important risk factor for cardiac, stroke, and kidney diseases (Shah et al., 2019). By 2025, it is estimated that the total number of patients with hypertension will rise to 1.56 billion worldwide (Xu et al., 2020). Studies have shown that various genetic and environmental factors, including dietary salt intake, lack of exercise, and alcohol consumption, also contribute to hypertension progression (Booth et al., 2012; Rust and Ekmekcioglu, 2016). Previous research on animal models and human subjects has shown that hypertension progression is also linked to gut microbiota dysbiosis (Jose and Raj, 2015; Miremadi et al., 2016). Moreover, alterations in the composition of the intestinal microbiota can result in the development of novel antihypertensive therapies. The various mechanisms underlying the relation between gut microbiota and hypertension have been proposed, although there is no definite understanding. The ratio of *Bacteroidetes* and *Firmicutes* within intestinal microbiota has been significantly associated with hypertension (Yang et al., 2015). Hypertensive animals and seven hypertensive patients reported an abundance of *Bacteroidetes* and *Firmicutes* in their gut microbiota as sequenced by 16S ribosomal RNA (Moghadamrad et al., 2015). Studies using angiotensin II-infused GF mice have shown that gut microbiota is involved in vascular dysfunction and hypertension induced by angiotensin II (Karbach et al., 2016).

Short-chain fatty acids play a crucial role in maintaining gut microbiome homeostasis and host immunity. Recent studies have found that SCFAs produced by gut microbiota is involved in modulating blood pressure (Kang and Cai, 2018). SCFAs have the potential to stimulate host G-protein-coupled receptor (GPR) pathways that affect the secretion of renin and blood pressure (Pluznick et al., 2013). In another study to investigate the correlation between serum metabolites and hypertension, it was found that lyxose levels (a by-product of intestinal microbial fermentation) were higher in patients with newly diagnosed hypertension compared to healthy controls (Hao et al., 2016). However, these findings are preliminary; it is essential to validate other environmental factors like the diet that might affect the gut microbiota.

Furthermore, a beneficial role of *Lactobacillus* in the regulation of blood pressure has been reported (Gómez-Guzmán et al., 2015). Recent studies and clinical trials demonstrate a close but complex inter-relationship between gut microbiota and hypertension. However, more studies involving human participants are needed to elaborate on the critical role of gut microbiota in hypertension and to demonstrate promising therapeutical approaches.

### Cardiovascular diseases

Even with the existing approaches in atherothrombosis prevention and treatment, heart disease is still a significant cause of death globally. It will constantly rise due to increased incidence in low and middle-income countries (Odutayo et al., 2016). In the pathophysiology and progression of CVDs, the intestine has also been involved, primarily due to decreased perfusion of the intestines leading to intestinal barrier dysfunction. The intestinal endothelial barrier is regulated by many mechanisms of a well well-balanced intestinal microbiota (Sabatino et al., 2015). Recently, due to accumulating evidence, intestinal microbiota has been studied as a contributing factor to heart disease and stroke (Tang et al., 2017; Leustean et al., 2018; Jayachandran et al., 2020). Emerging evidence has shown that gut dysbiosis was correlated with the production of many metabolites from intestinal microbiota and also fostered disruption of the function of the gut endothelial barrier.

Furthermore, an essential correlation between the amount of fecal gut microbiota and the intensity of intestinal permeability was identified in patients with CVDs (Pasini et al., 2016). In contrast, patients who had bacterial DNA in the peripheral blood had considerably high plasma levels of inflammatory markers, particularly highly sensitive C-reactive protein and interleukin-6 levels, compared to those who did not have bacterial DNA in their peripheral blood (Wang et al., 2012). Moreover, an increased abundance of *Streptococcus* and *Enterobacteriaceae* is linked with coronary artery disease (Jie et al., 2017). Patients with coronary artery disease have altered populations of the most prevalent bacterial species that make up the gut microbiota, with a decrease in Bacteroidetes and an increase in Firmicutes. Trimethylamine-N-oxide is a metabolite that plays an important role in atherosclerosis and can help predict cardiovascular risk (Ramírez-Macías et al., 2022).

Various mechanisms have been proposed to understand the crucial role of gut microbiota in the development and prevention of CVDs. Copies of bacterial genes coding for trimethylamine (TMA) lyase and atherosclerotic CVDs have also been found to be associated (Barrington and Lusis, 2017). TMA lyase contributes to the generation of trimethylamine-N-oxide (TMAO), a metabolite derived from the gut microbiota (Witkowski et al., 2022). TMAO has been shown to contribute to the development of cardiovascular atherosclerotic disease in animal studies and seems to be significantly linked in human studies, identifying the primary function that TMAO may perform in developing atherosclerotic CVD (Tang and Hazen, 2014; Jonsson and Bäckhed, 2017). Thus, a rapid increase in cardiovascular and metabolic disorders has concentrated on gut microbiota regulation as an effective treatment option.

### Diabetes mellitus

Diabetes mellitus causes a significant adverse effect on the health condition of human populations worldwide. Diabetes-related risk factors include aspects like a family history of diabetes, poor eating habits, and being overweight. Regarding the continuous rise of urbanization, shifts in diet, and the emergence of more unhealthy lifestyles, the growing incidence of diabetes is a global crisis. According to a report, about 463 million people globally reported diabetes in 2019, and future estimates predict that by 2045, the number of diabetic patients will exceed 700 million (Saeedi et al., 2019). Recent studies have demonstrated that the progression of diabetes is closely correlated to the alterations in the composition of intestinal microbiota (Sender et al., 2016; Gurung et al., 2020). Diet is among the key determinants of the composition of the intestinal microbiota and a significant causal factor in the development of diabetes (Meijnikman et al., 2018).

Given that the development and formation of the gut microbiota depend on the availability of nutrients, it is vitally important to demonstrate that metabolite production depends on food consumption. It has been found that, in response to a shift from a low-fat, plant polysaccharide-rich diet to a high-fat, high-sugar diet, the microbiome composition changed rapidly (Turnbaugh et al., 2009). Human eating patterns have evolved over the past few decades, with fats preferred over fibers; in response to recent eating habits, intestinal microbiota has also changed. Therefore, it was suggested that diabetes could be linked to the intestinal microbiota’s systematic alterations (Sircana et al., 2018).

It was observed in the diabetes prevention and prediction (DIPP) study that new-onset type-1 diabetes subjects had a distinct composition of gut microbiota compared to controls (Brown et al., 2011). It was found that mucin formation was caused by lactate and butyrate-producing bacteria in the control group to sustain gut integrity. In contrast, mucin synthesis was inhibited by non-butyrate producing lactate-utilizing bacteria contributing to autoimmunity of β-cells and type 1 diabetes (Brown et al., 2011). Also, an increase in the occurrence of *Akkermansia muciniphila* has been observed to be inversely related to the probability of developing type 1 diabetes (Hansen et al., 2012; Navab-Moghadam et al., 2017). *A. muciniphila* may is a potential probiotic in the treatment of type 1 diabetes. Many other studies have reported the variations in the composition of gut microbiota between type 1 diabetes and their matched health controls, illustrating the need for a better understanding of the function that these bacteria can play in the development of diabetes (Murri et al., 2013; Gülden et al., 2015).

It has been indicated that the influence of microbiota on type 2 diabetes can be mediated through mechanisms involving changes in the butyrate and incretins secretions (Nøhr et al., 2013; Baothman et al., 2016). In patients with type 2 diabetes, a study showed a moderate degree of intestinal microbial dysbiosis, a decrease in bacteria-producing universal butyrate, and an increase in opportunistic pathogens (Baothman et al., 2016). Other studies have also shown the significant influence of gut microbiota on type 2 diabetes pathways, including insulin signaling, inflammation, and glucose homeostasis (Baothman et al., 2016; Cani, 2018). However, more studies are needed to deeply understand the mechanisms and influential role of gut microbiota in the development of diabetes.

### Cancer

Cancer is the second most common cause of death globally (Fitzmaurice et al., 2017). Many factors significantly influence cancer risks, such as exposure to pathogens, UV radiation and toxic substances, diet, and lifestyle. However, the risk mainly depends on the dosage, the period, and the combination of such factors, along with the genetic background of the patient (Vivarelli et al., 2019). There is a growing interest in the characterization and functionality of intestinal microbiota due to its complicated relationship with the host (Tao et al., 2020). Different studies have indicated that abrogation or alteration of gut microbiota significantly contributes to developing colorectal carcinoma in genetic and carcinogenic tumorigenesis models (Arthur et al., 2012; Vivarelli et al., 2019). Metabolomics and metagenomics studies have demonstrated the dual role of gut microbiota in cancer risk reduction and tumor growth, and anti-cancer therapies (Bultman, 2014).

A greater abundance of *Bacteroides massiliensis* was found in patients with prostate cancer, while *Eubacterium rectale* and *F. prausnitzii* have been identified in comparatively less abundance, indicating the potential contribution of these specific microorganisms in the pathogenesis of prostate cancer (Chung et al., 2018). It has also been found that the gut microbiota is linked with the development of colorectal cancer, with *Fusobacterium nucleatum*, *Bacteroides fragilis*, and *Peptostreptococcus anaerobic* being identified in its development as important players (Hsieh et al., 2018). Gut bacteria, especially *F. nucleatum* and *Clostridium colicanis*, were proposed as indicative markers in gastric cancer’s carcinogenesis (Mehta et al., 2017). Recent studies have indicated that *F. nucleatum* can suppress the host’s immune response and upgrade cellular proliferation. In contrast, a diet rich in whole grains and dietary fiber have a lower risk of *F. nucleatum* positive cancer, indicating that the gut microbiome may be a significant mediator between dietary and colorectal cancer interactions (Hall et al., 2017). Various preclinical studies using GF mice have proposed the mechanism and considerable impact of gut microbiota on genesis and cancer progression (Arthur et al., 2012). A deeper understanding of the influential role of gut microbiota in the development of cancer has increased the interest in research for microbiome-based therapeutics in cancer treatment. However, more studies involving human participants are required to deeply understand the mechanism of gut microbiota in the development of cancer and its anti-carcinogenic characteristics.

### Inflammatory bowel diseases

Inflammatory bowel disease is a significant disease with the highest prevalence in western countries; its incidence has risen rapidly in newly industrialized countries in Asia, the Middle East, Africa, and South America (Kaplan and Ng, 2017). It is also imperative to examine the exact etiology and pathogenesis of IBD. Notable advancements have been achieved in identifying the development of IBD in the last few years. The most significant and clinically beneficial aspect of this advancement was the identification of gut microbiota as a crucial multifunctional inflammatory factor. Recently, the role of intestinal microbiota in the pathogenesis of IBD has been emphasized. Several lines of evidence indicate the essential part of the gut microbiota in intestinal inflammation. Most studies have demonstrated decreased intestinal microbiota diversity in patients with IBD (Willing et al., 2010; Matsuoka and Kanai, 2015). Significant decreases in *Firmicutes* and proteobacteria are the most important observations of altered composition of gut microbiota in patients with IBD. The decreased diversity of intestinal microbiota found in patients with IBD was primarily due to the reduction of *Firmicutes*. A decline in the *Clostridium leptum* groups, particularly *F. prausnitzii*, has been observed among Firmicutes (Wang et al., 2014). In biologically susceptible hosts, alterations of the gut microbiota have been associated with aberrant mucosal immune responses that result in a variety of intestinal and extraintestinal disorders, including IBD. As a result, restoring immunological homeostasis by modifying the gut microbiota is currently considered to be a potential therapeutic strategy to treat IBD patients (Facciotti, 2022).

The majority of discovered human pathogenic bacteria belong to the phylum Proteobacteria, which play an increasingly important role in IBD (Mukhopadhya et al., 2012). Analysis of microbial diversity shows a rise in the number of bacterial species belonging to this phylum, implying an active role in initiating chronic inflammation in patients with IBD (Hold et al., 2014). The abundance of *Ruminococcus gnavus* is also found to be higher in IBD (Örtqvist et al., 2019). Although more clinical studies are required to examine and deeply understand the mechanism through which gut microbiota contribute to IBD progression.

## Eubiosis and food

Dietary effects and influences on our gut microbiome are not new subjects of research. Food causes transient changes in the gut microbiota composition, which are primarily due to fish, meat, and fiber, which have long-term effects (Bajinka et al., 2020). More than two macronutrients can be found in one diet, which alters the gut microbiota while also altering metabolic output (Qiu et al., 2020). The positive benefits of dietary fiber on human metabolism have been explored and found to be significant. Dietary fiber has been shown to alter the microbiota and produce beneficial metabolites like butyrate (Silva et al., 2020). While a balanced nutritional diet is important for overall health, a diet high in fiber is particularly essential to maintain the diversity of the intestinal microbiota (Zhang et al., 2013).

Microbiota ferment complex undigested carbohydrates, also known as microbiota-accessible carbohydrates (MAC), leads to an increase in SCFA levels and, as a result, a positive health effect (Seo et al., 2020). These complex carbohydrates, which include resistant starch, oligosaccharides, and dietary fiber, can positively modulate a variety of gut microbes that are beneficial to health (Yang et al., 2020). Unsaturated plant-based fats in the diet reduce detrimental bacteria while increasing the abundance of *Bifidobacterium* and butyrate-producing bacteria (*Roseburia* and *Faecalibacterium*), all of which have been associated with positive health effects (Muralidharan et al., 2019). Micronutrients, in addition to macronutrients, may play a key role in gut reshaping, according to various studies (Ramos and Martín, 2021). All of these findings point to the importance of dietary factors as modulators of the microbial community, which can therefore have an impact on human physiology and disease processes.

## Conclusion

The crucial role of probiotics in health, disease, and nutrition has increased their scientific and marketing significance across the globe. The attention has been shifted from prospective studies to clinical trials to have a better understanding of how microbiota can interplay in human health and disease. Eubiosis is important in exerting the health endorsing benefits of probiotics. An unhealthy diet intake, such low intakes of fruits and vegetables intakes and overuse of antibiotics can result in dysbiosis. In nutshell, probiotics aid in the treatment of various infectious diseases, dysfunctions of the GI tract, and inflammatory disorders as well as in controlling obesity and diabetes. The advances in gut microbiota modeling and analysis will enhance our knowledge of how they influence health and disease, allowing us to adapt current and forthcoming therapeutic and preventive strategies. Understanding the specific roles played by the gut microbiome in our growth and development, as well as how it functions in health and disease, holds the potential to improve many parts of our daily lives, from improving the formula for infants to offering new approaches in fighting obesity and cancer, among others. As gut microbiota is a complex topic, future research should focus on multidisciplinary approaches, taking into consideration recent innovations in various scientific fields.

## Author contributions

MA and RA: conceptualization and writing—original draft preparation. MA, FS, YS, MH, RR, AH, AR, MP, JL, and CS: writing—review and editing. RA: supervision. All authors read and agreed to the final version of the manuscript.
